# A Novel Control Method of *Enterococcus faecalis* by Co-Treatment with Protamine and Calcium Hydroxide

**DOI:** 10.3390/pharmaceutics15061629

**Published:** 2023-05-31

**Authors:** Yu Abe, Michiyo Honda

**Affiliations:** Department of Applied Chemistry, School of Science and Technology, Meiji University, 1-1-1, Higashimita, Tama-ku, Kawasaki 214-8571, Japan; y.abe1825@gmail.com

**Keywords:** protamine, root canal treatment, *Enterococcus faecalis*, antimicrobial peptide

## Abstract

*Enterococcus faecalis* (*E. faecalis*), a gram-positive facultative anaerobic bacterium, is likely to survive root canal treatment because of its extremely high alkaline tolerance, which may contribute to the refractory nature of apical periodontitis (AP). In this study, protamine was combined with calcium hydroxide to evaluate its efficacy in killing *E. faecalis*. First, the antibacterial activity of protamine against *E. faecalis* was investigated. Protamine reduced the *E. faecalis* growth rate at concentrations above the MIC (250 μg/mL), but was not bactericidal at any of the concentrations tested. Next, we investigated the calcium hydroxide tolerance of *E. faecalis*, using a 10% 310 medium, adjusted for pH by adding a calcium hydroxide solution. The results showed that *E. faecalis* could survive and proliferate in alkaline environments up to pH 10. However, the complete killing of *E. faecalis* was observed when protamine (250 μg/mL) was added. In addition, compared with treatment with protamine and calcium hydroxide alone, membrane damage and internalization of protamine into the cytoplasm of *E. faecalis* were enhanced. Therefore, the synergistic increase in antibacterial activity may be related to the action of both antimicrobial agents on the cell membrane. In conclusion, co-treatment with protamine and calcium hydroxide seems to be very effective in sterilizing *E. faecalis*, and has the potential to provide a novel control method against *E. faecalis* for root canal treatment.

## 1. Introduction

Apical periodontitis (AP) is an inflammatory dental disease caused by microbial infection of the root canal [1], which often requires root canal treatment to control the infection and eliminate inflammation [2]. Root canal treatment has been shown to be beneficial for achieving aseptic root canals [3,4]. However, there is still no assurance of a complete cure after the first treatment [5], and many cases become refractory. The most likely reason for this poor prognosis is the presence of uncontrolled surviving microorganisms in the root canal [2]. The gram-positive facultative anaerobic bacterium *Enterococcus faecalis* (*E. faecalis*) is frequently detected in root canals with failed treatment [6,7,8]. This suggests that the root canal treatment was effective against many other bacteria, but ineffective against *E. faecalis*, and the presence of these bacteria may be one of the factors that make AP difficult to treat. Therefore, it would be indispensable to investigate more effective methods for controlling *E. faecalis* in root canals, to improve the success rate of AP treatments.

The purpose of root canal treatment is to eradicate pathological flora from the root canal system and achieve complete tooth asepsis [9]. Calcium hydroxide has been reported to be effective in eradicating residual bacteria because of its ability to provide a highly alkaline environment within the tooth [3]. In contrast, *E. faecalis* has an extremely high alkaline tolerance, due to its unique membrane durability, impermeability, and proton pump mechanism [10,11]. Therefore, it is highly likely to survive in the strongly alkaline environment provided by calcium hydroxide. An in vitro study reported that an alkaline environment of pH 11.5 or higher is necessary for complete sterilization [11]. Unlike the in vitro environment, the actual root canal environment is a complex mixture of inorganic and organic components, such as hydroxyapatite, a major component of dentin, and albumin, contained in the exudate. Previous studies reported that these root canal-forming components reduce the antimicrobial activity of calcium hydroxide [12]. Probably, the pH conditions necessary for *E. faecalis* disinfection are not met deep in the dentin tubules because of the buffering effect of these dentin tubules. [11,13]. Thus, *E. faecalis* survival may be promoted in real root canal environments. To improve the success rate of root canal treatment, it is essential to consider more effective treatment methods of calcium hydroxide against *E. faecalis*.

In this study, we focused on antimicrobial peptides (AMP), which have recently attracted attention as novel antimicrobial agents [14,15,16]. Protamine is a strongly basic and cationic AMP, rich in arginine residues derived from salmon milt, with a molecular weight of approximately 5 kDa, and an isoelectric point of 12–13. The typical characteristics include high biocompatibility when used as a food preservative, antimicrobial activity that reaches its maximum in the basic range, a high isoelectric point that does not aggregate even in the basic range, and resistance to thermal denaturation that can withstand autoclave sterilization [17]. In addition, it has recently been reported to have excellent antimicrobial activity against oral microorganisms [18], which increases synergistically when used in combination with existing antimicrobial agents [19]. Thus, protamine can potentially be used in the dental field, which requires biocompatibility, antibacterial activity, and stability; however, the details remain unclear. In this study, we investigated the antimicrobial properties of protamine alone against *E. faecalis*, and evaluated the efficacy of co-treatment with protamine and calcium hydroxide in killing *E. faecalis*.

Our study provides new insights into the bacterial control, especially *E. faecalis*, a type of bacteria involved in AP, and proposes a novel method for root canal treatment.

## 2. Materials and Methods

### 2.1. Culture Medium

A 310 medium (NBRC) was used to culture the microorganisms. In this study, assuming a poor nutritional environment after endodontic cleaning, a 310 medium, diluted with ultrapure water to a final concentration of 10%, was used for various evaluation tests.

### 2.2. Microorganisms

The strain of *E. faecalis* purchased from NBRC (NBRC No. 100481) was used in this study. *E. faecalis* was restored by 310 medium and cultured anaerobically at 37 °C using an anaerobic jar (Mitsubishi gas chemical, Tokyo, Japan) and AnaeroPack^®^ Kenki (Mitsubishi gas chemical, Tokyo, Japan). The culture was stored at −80 °C in 15% glycerol.

### 2.3. Antimicrobial Agents

Protamine was supplied by Maruha Nichiro Corporation (Tokyo, Japan). Protamine (0.5 g) was dissolved in 50 mL of ultrapure water (10,000 μg/mL). The solution was sterilized in an autoclave (121 °C, 20 min), diluted appropriately, and used for the various evaluation tests.

To prepare the calcium hydroxide solution, supersaturated amounts of calcium hydroxide powder (FUJIFILM Wako Pure Chemical, Osaka, Japan) were dissolved in ultrapure water. The solution was centrifuged at 8000 rpm for 10 min. The supernatant was used for evaluation tests.

### 2.4. Evaluation of the Antimicrobial Activity of Protamine against E. faecalis

#### 2.4.1. Antimicrobial Susceptibility Testing of *E. faecalis* to Protamine

The susceptibility of *E. faecalis* to protamine was evaluated by alamarBlue^®^ assay and colony count assays. Protamine (20 μL), bacterial suspension (5 × 10^5^ CFU/mL) diluted by 10% 310 medium, and 20 μL of alamarBlue^®^ solution (Thermo, Waltham, MA, USA) were added to 96 well plates. Testing was performed using appropriate controls. After incubation for 24 h at 37 °C, the color of the solution changed. The minimum inhibitory concentration (MIC) was determined as the lowest concentration at which no color change occurred (red indicates growth and blue indicates no growth). For concentrations above the MIC, 100 μL of the suspension from each well was plated onto a 310 agar plate, incubated for 24 h at 37 °C in an anaerobic environment, and then observed for the presence of colonies. Minimum bactericidal concentration (MBC) was determined as the lowest concentration that resulted in no growth on agar plates.

#### 2.4.2. Evaluation of Growth Inhibition Ability of Protamine against *E. faecalis*

To evaluate the growth-inhibitory ability of protamine against *E. faecalis*, we treated planktonic cells with protamine, and determined changes in viable bacterial counts over time using a colony count assay. *E. faecalis* was incubated anaerobically overnight in 310 mL of medium, and the cell density was adjusted to 5 × 10^6^ CFU/mL. Antimicrobial treatment was performed by mixing 100 μL of the bacterial suspension, 100 μL of Protamine (2500, 5000, 10,000 μg/mL), and 800 μL of sterile water. After incubation of these samples at 37 °C for 1, 2, 4, 8, and 24 h, 100 μL of suspension from each sample was plated to a 310 agar plate, and the viable bacterial count was determined by colony count.

### 2.5. Evaluation of the Antimicrobial Activity of Calcium Hydroxide against E. faecalis

#### 2.5.1. Evaluation Test of Calcium Hydroxide Tolerance of *E. faecalis*

To evaluate the calcium hydroxide tolerance of *E. faecalis*, a calcium hydroxide solution was added to 10% 310 medium, and the planktonic cells were incubated for 24 h in an arbitrary alkaline environment. Viable bacterial counts were determined using the colony count assay. *E. faecalis* was incubated anaerobically overnight in 310 medium. The suspension was replaced with 10% 310 medium, and the cell density was adjusted to 5 × 10^7^ CFU/mL. Antimicrobial treatment was conducted by mixing 10 μL of bacterial suspension and 990 μL of 10% 310 medium, with the pH adjusted to 8, 10, or 12 by adding a calcium hydroxide solution. After incubation of these samples at 37 °C for 24 h, 100 μL of suspension from each sample was plated to a 310 agar plate, and the viable bacterial count was determined by colony count.

#### 2.5.2. Effect of Treatment with Calcium Hydroxide on the Metabolic Activity of *E. faecalis*

To evaluate the effect of calcium hydroxide treatment on the metabolic activity of *E. faecalis*, planktonic cells were incubated in an arbitrary alkaline environment, and changes in viable bacterial count over time were determined by a colony count assay. *E. faecalis* was incubated anaerobically overnight in a 310 medium. The suspension was replaced with 10% 310 medium, and the cell density was adjusted to 5 × 10^7^ CFU/mL. Antimicrobial treatment was conducted by mixing 10 μL of the bacterial suspension and 990 μL of a 10% 310 medium, with the pH adjusted to 8 and 10 by adding calcium hydroxide solution. After incubation of these samples at 37 °C for 2, 4, 8, and 24 h, 100 μL of suspension from each sample was plated to a 310 agar plate, and the viable bacterial count was determined by colony count.

### 2.6. Evaluation of the Antimicrobial Activity of Co-Treatment with Protamine and Calcium Hydroxide

#### 2.6.1. Evaluation of Antimicrobial Activity

To evaluate the effectiveness of co-treatment with protamine and calcium hydroxide in killing *E. faecalis*, planktonic cells were co-treated with protamine and calcium hydroxide, and the viable bacterial count was determined using a colony count assay. *E. faecalis* was incubated anaerobically overnight in a 310 medium. The suspension was replaced with a 10% 310 medium, and the cell density was adjusted to 5 × 10^7^ CFU/mL. Antimicrobial treatment was conducted by mixing 10 μL of the bacterial suspension and 990 μL of a 10% 310 medium, with the pH adjusted to 8 and 10 by adding calcium hydroxide solution. If necessary, protamine was added to 990 μL of a 10% 310 medium, to a final concentration of 250 μg/mL. After incubation of these samples at 37 °C for 1 or 24 h, 100 μL of suspension from each sample was plated in a 310 agar plate, and the viable bacterial count was determined by colony count.

#### 2.6.2. Evaluation of the Effect on the Surface Structure of *E. faecalis*

To evaluate the effect of co-treatment with protamine and calcium hydroxide on the surface structure of *E. faecalis*, we observed the bacteria after treatment using scanning electron microscopy (SEM) after treatment, as described in Section 2.6.1. The samples were washed once with PBS and fixed with 2.5% glutaraldehyde. After 2 h, the samples were washed once with PBS and dehydrated stepwise with ethanol in the following order: 50, 70, 90, 95, 99, and 100%. Samples resuspended in 100% ethanol were dropped onto Thermanox^®^ Plastic Coverslips (Thermo Fisher Scientific, Waltham, MA, USA) and air-dried overnight. They were then coated with gold and observed by SEM (VE-9800, KEYENCE, Osaka, Japan).

### 2.7. Investigation of Antimicrobial Mechanism of Action

#### 2.7.1. Observation of Cell Membrane Damage Using Live/Dead Staining

To evaluate the effect of co-treatment with Protamine and calcium hydroxide on the cell membrane of *E. faecalis*, we examined the presence of cell membrane damage by Live/Dead^®^ *Baclight*^™^ Bacterial Viability Kit (Thermo, Waltham, MA, USA). *E. faecalis* was incubated anaerobically overnight in 310 medium. The suspension was replaced with 10% 310 medium, and the cell density was adjusted to 2 × 10^9^ CFU/mL. We mixed 50 μL of the bacterial suspension and 950 μL of a 10% 310 medium, with pH adjusted to 8 and 10 by adding a calcium hydroxide solution (hereinafter called “Suspension A”). Antimicrobial treatment was conducted by mixing 324.97 μL of Suspension A, 8.33 μL of protamine (final concentration; 250 μg/mL), 0.5 μL of SYTO9, and 0.5 μL of Propidium Iodide (PI). After incubation of these samples at 37 °C for 1 h, they were washed three times with PBS, and observed by an all-in-one fluorescence microscope (BZ-X710, KEYENCE, Osaka, Japan).

#### 2.7.2. Investigation of Localization Sites of Protamine in Bacteria

To investigate the relationship between cell membrane damage and localization site of protamine, and the relationship between the localization site of protamine and antimicrobial activity, fluorescence microscopy was conducted using N-terminal Carboxyfluorescein hydrate (FAM), labeled protamine peptide (FAM-PP), synthesized by SCRUM Corporation (Tokyo, Japan). *E. faecalis* was incubated anaerobically overnight in a 310 medium. The suspension was replaced with a 10% 310 medium, and the cell density was adjusted to 2 × 10^9^ CFU/mL. We mixed 50 μL of bacterial suspension and 950 μL of 10% 310 medium, with pH adjusted to 8 and 10 by adding a calcium hydroxide solution (hereinafter called “Suspension B”). The antimicrobial treatment was performed by mixing 324.97 μL of Suspension B, 8.33 μL of FAM-PP (final concentration: 250 μg/mL), and 0.5 μLof P. After incubation of these samples at 37 °C for 1 h, they were washed three times with PBS, and observed by an all-in-one fluorescence microscope (BZ-X710, KEYENCE, Osaka, Japan).

In this study, various ratios were calculated using the following formulae:

Viability (%) = (viable bacterial count treated with antimicrobial agent/viable bacterial count without antimicrobial treatment) × 100

Cell membrane Localization (%) = (bacterial count of cell membrane localization/total bacterial count) × 100

Cell membrane internalization (%) = (bacterial count of cell membrane internalization/total bacterial count) × 100

Cell membrane damage (%) = (bacterial count of cell membrane damage/total bacterial count) × 100

## 3. Results

### 3.1. Antimicrobial Activity of Protamine against E. faecalis

#### 3.1.1. Antimicrobial Susceptibility Testing of *E. faecalis* to Protamine

To examine the antimicrobial susceptibility of *E. faecalis* to protamine, MIC and MBC were determined using alamarBlue^®^ assay and colony count assays, respectively. Table 1 lists the MIC and MBC values. Treatment with protamine inhibited the normal growth of *E. faecalis* (MIC: 250 μg/mL), although it was not bactericidal at any of the concentrations tested (MBC: >1000 μg/mL).

#### 3.1.2. Growth Inhibition Ability of Protamine against *E. faecalis*

**Figure 1:**
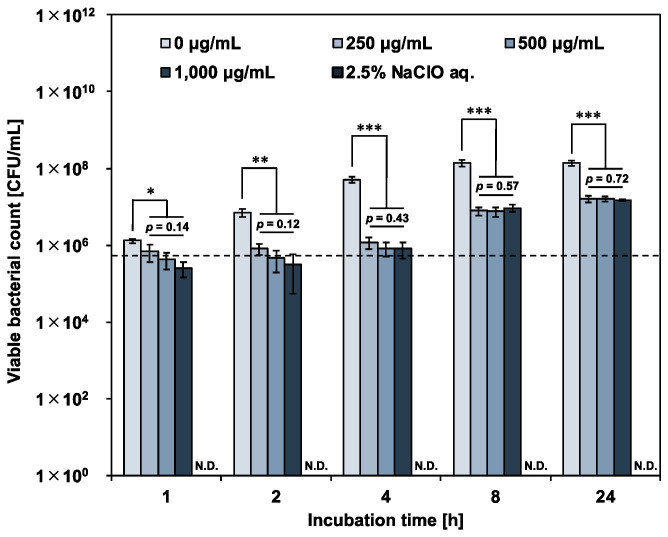
Effect of protamine treatment on *E. faecalis* growth rate. Viable bacteria were quantified using a colony-counting assay. Negative control: sterile water; positive control: 2.5% sodium hypochlorite solution. Dotted line represents initial bacterial counts. Error bars indicate the standard deviation of the mean (*n* = 3). Asterisks denote statistical significance: * *p* < 0.05, ** *p* < 0.01, *** *p* < 0.001 (Student’s *t*-test). Protamine concentration dependence investigated using one-way ANOVA. “N.D.” indicates not detected.

To evaluate the growth inhibitory ability of protamine against *E. faecalis*, cells were treated with protamine at concentrations above the MIC, and changes in viable bacterial count over time were determined by a colony count assay. Figure 1 shows the changes in the viable bacterial count after treatment. The growth rate of *E. faecalis* treated with protamine was significantly lower than that of the negative control (sterile water). However, an increase in the viable bacterial count was observed with prolonged incubation. The bactericidal activity of the positive control (2.5% NaClO aq.) was not detectable. These results suggest that a single treatment of protamine is difficult to control *E. faecalis*. No concentration dependence was observed in the growth inhibitory ability of protamine against *E. faecalis*. Therefore, protamine at a concentration of 250 μg/mL (MIC) was used for subsequent tests.

### 3.2. Antimicrobial Activity of Calcium Hydroxide against E. faecalis

**Figure 2:**
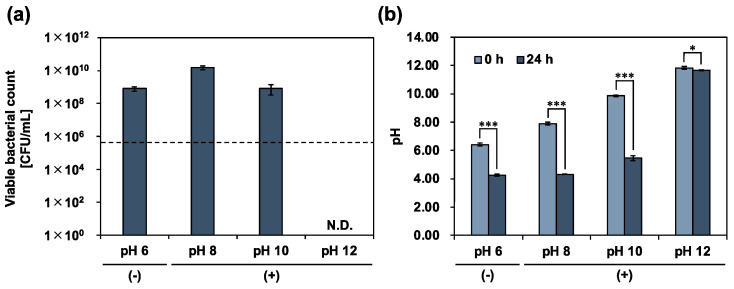
Calcium hydroxide tolerance of *E. faecalis*. (**a**) *E. faecalis* was incubated for 24 h in a 10% 310 medium, with varying pH adjusted by calcium hydroxide addition. Viable bacteria quantified by colony-count assay. (−): no calcium hydroxide, (+): with calcium hydroxide. Dotted line represents initial bacterial counts. (**b**) pH of culture medium measured at start and end of incubation. Error bars indicate the standard deviation of the mean (*n* = 3). Asterisks denote statistical significance: * *p* < 0.05, *** *p* < 0.001 (Student’s *t*-test). “N.D.” indicates not detected.

**Figure 3:**
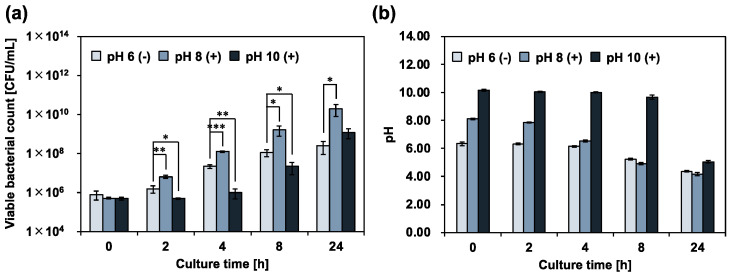
Effect of calcium hydroxide treatment on *E. faecalis* metabolic activity. (**a**) *E. faecalis* was treated in 10% 310 medium, with varying pH adjusted using calcium hydroxide. Viable bacteria quantified by colony-count assay. (−): no calcium hydroxide, (+): with calcium hydroxide. (**b**) pH of culture medium measured at start and end of incubation. Error bars indicate the standard deviation of the mean (*n* = 3). Asterisks denote statistical significance: * *p* < 0.05, ** *p* < 0.01, *** *p* < 0.001 (Student’s *t*-test).

To examine the calcium hydroxide tolerance of *E. faecalis*, a calcium hydroxide solution was added to 10% 310 medium, and the planktonic cells were incubated for 24 h in an arbitrary alkaline environment. The viable bacterial counts were determined using a colony-count assay (Figure 2a). The pH of the culture medium was simultaneously measured at the same time (Figure 2b). In an alkaline environment (pH 12), complete death of *E. faecalis* was observed, and the pH of the culture medium decreased slightly. In contrast, in alkaline environments (pH 8 and 10), *E. faecalis* survived. Cell growth was also observed and compared with the initial number of bacteria. After incubation, the pH of the culture medium decreased to the same level as that of the control (without calcium hydroxide; pH 6 (−)). *E. faecalis*, a lactic acid bacterium, produces lactic acid via homofermentation [20]. Therefore, the decrease in the pH of the culture medium was due to the metabolic activity of *E. faecalis* in alkaline environments at pH 8 and 10. These results show that *E. faecalis* maintains its metabolic activity, even in a strongly alkaline environment, at pH 10. We further examined the effect of calcium hydroxide treatment on the metabolic activity of *E. faecalis* by measuring the viable bacterial count and the pH of the culture medium over time. The evaluation was limited to the alkaline range (up to pH 10), where metabolism was possible. *E. faecalis* growth was significantly enhanced in the alkaline environment at pH 8 (Figure 3). A previous study reported that the optimum pH for *E. faecalis* growth ranges from 6 to 8 [21]. Our data can be explained by the fact that the samples were in an optimum pH environment compared with the control (pH 6 (−). In contrast, the growth of *E. faecalis* was significantly inhibited for up to 8 h in an alkaline environment at pH 10. During the suppression of bacterial growth, the rate of decrease in the pH of the culture medium was the lowest among all the samples. Our results indicated the involvement of calcium hydroxide in the metabolic activities of *E. faecalis*.

### 3.3. Antimicrobial Activity of Co-Treatment with Protamine and Calcium Hydroxide

**Figure 4:**
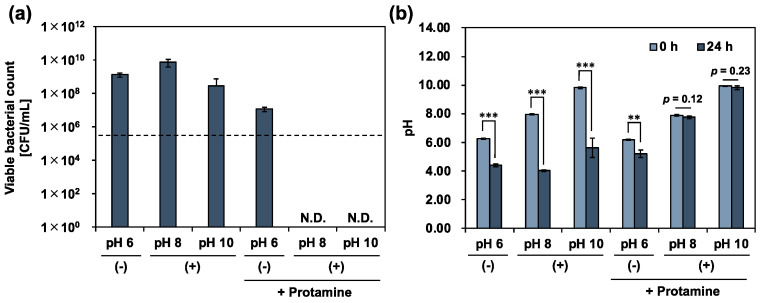
The antimicrobial activity of co-treatment with protamine and calcium hydroxide for 24 h against *E. faecalis*. (**a**) Viable bacteria quantified by colony-count assay after 24 h of co-treatment in 10% 310 medium. (−): no calcium hydroxide, (+): calcium hydroxide added, + protamine: protamine added (250 μg/mL). Dotted line represents initial bacterial count. (**b**) pH of culture medium measured at the beginning and end of the treatment. Error bars indicate the standard deviation of the mean (*n* = 3). Asterisks denote statistical significance: ** *p* < 0.01, *** *p* < 0.001 (Student’s *t*-test). “N.D.” indicates not detected.

**Figure 5:**
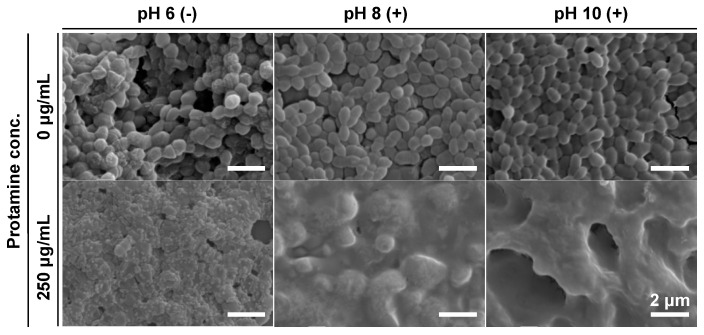
Morphological observation after 24 h of co-treatment with protamine and calcium hydroxide against *E. faecalis*. Treatment was performed in a 10% 310 medium. (−): no calcium hydroxide, (+): calcium hydroxide added.

**Figure 6:**
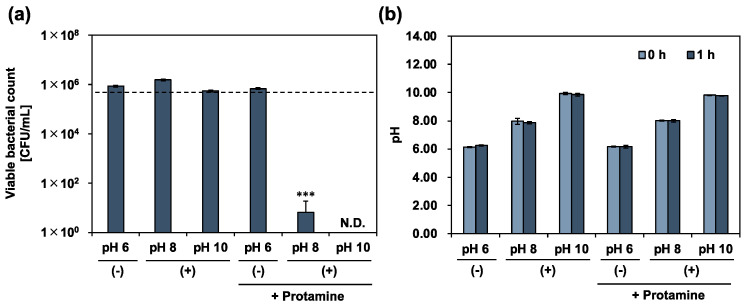
The antimicrobial activity of co-treatment with protamine and calcium hydroxide for 1 h against *E. faecalis*. (**a**) Viable bacteria quantified by colony-count assay after 1 h of co-treatment in 10% 310 medium. (−): no calcium hydroxide, (+): calcium hydroxide added, + protamine: protamine added (250 μg/mL). Dotted line represents initial bacterial count. (**b**) pH of culture medium measured at the beginning and end of the treatment. Error bars indicate the standard deviation of the mean (*n* = 3). Asterisks denote statistical significance: *** *p* < 0.001 (Student’s *t*-test). “N.D.” indicates not detected.

**Figure 7:**
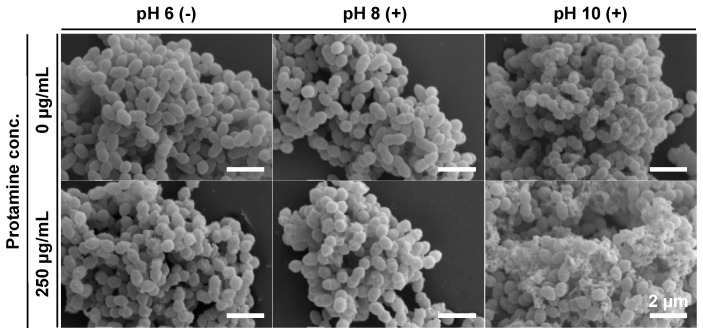
Morphological observation after 1 h of co-treatment with protamine and calcium hydroxide against *E. faecalis*. Treatment was performed in a 10% 310 medium. (−): no calcium hydroxide, (+): calcium hydroxide added.

The use of protamine or calcium hydroxide alone was valid, but not sufficient, for the control of *E. faecalis*. We then co-treated planktonic cells with protamine and calcium hydroxide. To evaluate the effectiveness of co-treatment with protamine and calcium hydroxide in killing *E. faecalis*, planktonic cells were co-treated with protamine and calcium hydroxide, and the viable bacterial count was determined using a colony-count assay. After 24 h of treatment, no death of *E. faecalis* was observed after a single dose, as shown in Figure 2. In contrast, the co-treatment completely killed *E. faecalis* (Figure 4a). Bacteriolytic activity was observed for *E. faecalis*, unable to maintain its surface structure (Figure 5). Additionally, no decrease in the pH of the culture medium was observed (Figure 4b). *E. faecalis* may have died within a relatively short time because of the inhibition of its metabolic activity. Therefore, we reduced the treatment time from 24 to 1 h, using the same protocol, and performed an additional test. In the alkaline environment at pH 10, *E. faecalis* was completely killed, as in the 24 h treatment (Figure 6a). Many *E. faecalis* strains with disrupted surface structures were observed (Figure 7). Even in an alkaline environment (pH 8), over 99.99% mortality was observed under the co-treatment conditions (Figure 6a). These results suggest that co-treatment with protamine and calcium hydroxide is effective in killing *E. faecalis*.

### 3.4. Investigation of Antimicrobial Mechanism of Action

Co-treatment with protamine and calcium hydroxide synergistically increased the antimicrobial activity and enabled effective disinfection of *E. faecalis*. In this study, to gain insight into the underlying mechanism, we performed two investigations focusing on the localization sites of protamine in bacteria.

**Figure 8:**
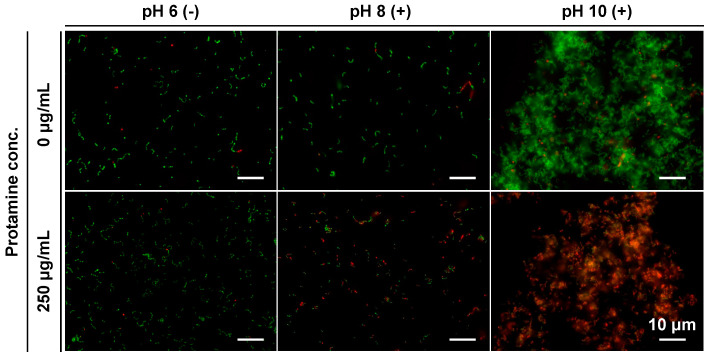
Cell membrane damage in *E. faecalis* observed after 1 h of co-treatment with protamine and calcium hydroxide. Cell membrane damage was evaluated by Live/Dead staining. Green fluorescence (SYTO9) represents intact cell membranes, while red fluorescence (propidium iodide; PI) indicates cell membrane damage. Treatment was conducted in 10% 310 medium. (−): no calcium hydroxide, (+): calcium hydroxide added.

First, Live/Dead staining was used to evaluate the effect of the co-treatment on the cell membranes of *E. faecalis*. The images showed that green fluorescence, indicating a normal cell membrane, was predominant when only protamine and calcium hydroxide were used (Figure 8). In contrast, under the co-treatment conditions, red fluorescence, indicating cell membrane damage, was predominant. These data suggest that cell membrane damage enhancement by cotreatment with protamine and calcium hydroxide may be responsible for the increased antimicrobial activity.

**Figure 9:**
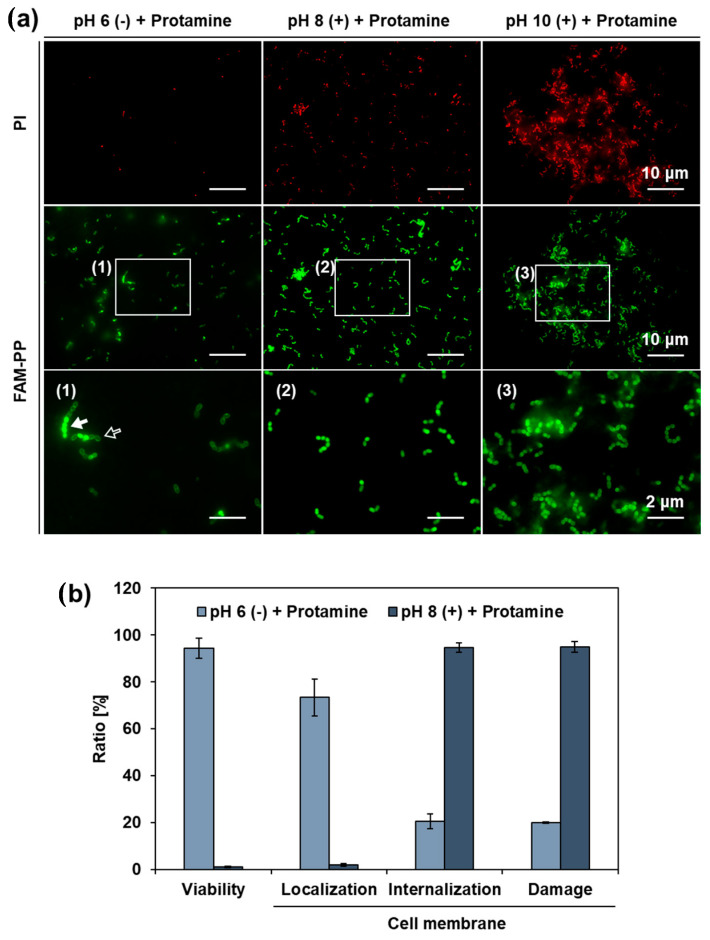
Localization sites of protamine in *E. faecalis* observed after 1 h of co-treatment with FAM-PP and calcium hydroxide. (**a**) Cell membrane damage was evaluated using PI staining, and localization sites of protamine was evaluated using FAM-PP. (1), (2), and (3) show higher magnification views of the area indicated by the white box. Red fluorescence represents PI, and green fluorescence represents FAM-PP. Treatment conducted in 10% 310 medium. (−): no calcium hydroxide, (+): calcium hydroxide added. The white arrow indicates protamine penetration through the cell membrane, while the black arrow indicates protamine localization on the cell membrane surface. (**b**) Ratios quantified by image analysis. Five randomly selected fields of view were observed (mean number of bacteria per field of view: 197.2), and the mean value was used for ratio calculations. Error bars indicate the standard deviation of the mean (*n* = 3).

Second, fluorescence microscopy, using FAM-PP and PI, was used to investigate the relationship between cell membrane damage and the localization sites of protamine, and the relationship between the localization sites of protamine and antimicrobial activity. Cotreatment with protamine and calcium hydroxide increased the percentage of protamine that permeated the cell membrane (Figure 9a). This internalization was more pronounced in the pH 8 (+) + protamine group, and showed a slightly decreasing trend at pH 10 (+) + protamine. Many bacteria with collapsed surface structures were observed in pH 10 (+) + protamine (Figure 7). Therefore, it is possible that protamine, which penetrates the cell membrane, leaks out, together with the inclusions. Thus, we quantified the various ratios shown in Figure 9b by comparing pH 6 (−) + protamine and pH 8 (+) + protamine. As a result, the percentage of cell membrane damage, which was 20.0 ± 0.270% in the protamine single treatment, increased to 95.0 ± 2.22% when protamine was combined with calcium hydroxide. In addition, while 73.4 ± 7.83% of the protamine worked in the protamine single treatment, localized at the cell membrane surface, this percentage decreased to 1.8 ± 0.656% in the sample combined with calcium hydroxide, and, instead, 94.7 ± 1.99% of the protamine penetrated the cell membrane. These results suggest that disruption of the cell membrane by co-treatment with protamine and calcium hydroxide may have promoted the permeation of protamine into the cytoplasm and synergistically inhibited the metabolism of *E. faecalis*.

## 4. Discussion

The use of calcium hydroxide as an intracanal medication has been reported to be beneficial for endodontic treatment because of its excellent antibacterial activity, as well as its ability to inhibit exudate, induce hard tissue, and dissolve organic material [3,4]. Nevertheless, a complete cure for AP during initial treatment has not yet been achieved [5]. This is because of the existence of microorganisms that are tolerant to the antimicrobial agents used for treatment. *E. faecalis* is recognized as one of these microorganisms. *E. faecalis* is highly tolerant of calcium hydroxide, and it has been reported that a highly alkaline environment of pH 11.5 is required for its complete mortality [7,11]. Calcium hydroxide ionizes into calcium and hydroxide ions in the solution, creating a highly alkaline environment of approximately pH 12 in the tooth [3]. However, due to the buffering effect of dentin, the pH does not usually exceed 11 throughout the root canal, especially in the dentin tubules [13]. *E. faecalis* invading the dentinal tubules is likely to survive treatment with calcium hydroxide, and is frequently detected in the root canals of patients with refractory AP [6,7]. Therefore, a new solution is needed to improve the success rate of root canal treatment and ensure a complete cure for AP. In this study, we selected protamine from AMP, which has recently attracted attention as a new antimicrobial agent, and applied it to root canal treatment, in combination with calcium hydroxide. Previous studies have shown that protamine may synergistically increase antimicrobial activity when used in combination with other antimicrobial agents, allowing for more effective microbial control [18,19]. In addition, the antimicrobial activity of AMP is generally thought to be due to electrostatic interactions between microorganisms and AMP [22,23]. Protamine has a very high isoelectric point of 12–13, and approximately 70% of the protamine molecule is composed of arginine residues, which allows it to carry a strong positive charge, even in highly alkaline environments. Therefore, protamine may have the potential to establish a good relationship with calcium hydroxide and enable a more effective control of *E. faecalis*. Protamine treatment at concentrations above the MIC (250 μg/mL) significantly reduced the growth rate of *E. faecalis.* However, no bactericidal activity was observed at any of the concentrations used in this study, and no concentration-dependent antibacterial activity was observed. Previous studies investigating the MIC and MBC of protamine against various oral bacteria have shown that the MBC of *E. faecalis* is extremely high compared to that of other oral bacteria [18]. Therefore, it is difficult to completely control *E. faecalis* with protamine alone.

Next, we investigated the calcium hydroxide tolerance of *E. faecalis*. *E. faecalis* was suspended in a 10% 310 medium, adjusted to pH 8, 10, and 12 by adding a calcium hydroxide solution, and incubated for 24 h. The results showed that an alkaline environment at pH 12 completely killed *E. faecalis*; however, its survival and growth were observed in alkaline environments up to pH 10. Previous studies have reported that a strongly alkaline environment with a pH of 11.5 or higher is necessary for the complete mortality of *E. faecalis* [7], and the results of this study provide positive data for previous studies.

It is not clear whether calcium hydroxide has any effect on *E. faecalis*, based on the observation of one point after 24 h in a sample with a pH of up to 10. Therefore, we limited the conditions up to pH 10, where biological activity could be maintained, and observed changes in viable counts over time. Growth was promoted in an alkaline environment at pH 8, whereas that was suppressed in an alkaline environment at pH 10. In relation to this result, previous studies have reported that *E. faecalis* can maintain an internal pH of 7.5–8.5 within the pH range of 8–10, while other bacteria have equal values of external and internal pH [10]. Therefore, the alkaline environment provided by calcium hydroxide is believed to alter the cytoplasmic pH of *E. faecalis* and affect its metabolism.

Finally, we limited the conditions to an alkaline range of up to pH 10, where biological activity could be maintained, and evaluated the efficacy of co-treatment with protamine and calcium hydroxide in the disinfection of *E. faecalis*. The results showed that 24 h of co-treatment with protamine and calcium hydroxide completely killed *E. faecalis* under alkaline conditions at pH 8 and 10. In addition, the 1 h co-treatment also showed complete mortality at pH 10, and over 99.9% mortality even at pH 8. These results suggested that co-treatment with protamine and calcium hydroxide was effective in killing *E. faecalis*. In clinical application, calcium hydroxide is considered the first choice of root canal treatment. However, it is difficult to control microorganisms, especially *E. faecalis*, using only calcium hydroxide. Additionally, a highly alkaline environment by calcium hydroxide damages the bacteria, but it also has adverse effects on humans. In contrast, our approach can provide a safe and less toxic environment, and has potential clinical applications.

Based on these results, we investigated the background mechanisms of the effect of co-treatment with protamine and calcium hydroxide on the synergistic increase in antimicrobial activity. Previous studies have reported the importance of cell membrane permeabilization in the antimicrobial activity of protamine [19]. In this study, we investigated the appearance of cell membranes and the protamine localization sites after treatment, using fluorescence microscopy with live/dead staining and FAM-PP. The results showed that 1 h of treatment with protamine or calcium hydroxide alone did not damage the cell membranes of *E. faecalis*. It has been reported that the mechanisms of action of calcium hydroxide are as follows: (i) disruption of cell membranes by hydroxide ions, (ii) increase in cytosolic pH due to hydroxide ion influx, (iii) metabolic inhibition by enzyme denaturation, and (iv) destruction of DNA [3,11]. Therefore, the survival of *E. faecalis* in an alkaline environment up to pH 10 is likely to depend mainly on the durability of its cell membrane. A previous study expressed the same view [10]. In contrast, under the condition of co-treatment with protamine and calcium hydroxide, the cell membrane was damaged, and it was also found that protamine was localized in the cytoplasm at a high rate. Although the exact mechanism of the antimicrobial action of protamine has not yet been clarified, it has been reported that protamine adheres to negatively charged bacterial surfaces, induces leakage of K^+^, ATP, and other intracellular enzymes [24], inhibits energy transfer and nutrient uptake functions by blocking the proton drive [25], increases cell membrane permeability, and promotes chlorhexidine transport into the cytoplasm [18]. Therefore, the dramatic increase in antimicrobial activity by co-treatment with protamine and calcium hydroxide may be due to the following mechanisms: (i) protamine disrupts the durability and impermeability of *E. faecalis* cell membranes; (ii) membrane disruption is promoted by hydroxide ions; (iii) this damage promotes protamine internalization into the cytoplasm; and (iv) protamine and hydroxide ions act synergistically in the cytoplasm. However, further studies are needed to determine the antimicrobial mechanisms of protamine after its entry into the cells.

In both experiments, more bacteria were observed at pH 10 than at other pH values. It is clear, from the results shown in Figure 6, that this was not an effect of bacterial growth. Therefore, two factors are possible: (i) the addition of calcium hydroxide promoted protein aggregation in the culture medium, which, in turn, promoted the aggregation of bacteria; and (ii) because numerous bacteria with disrupted surface structures were observed under the pH 10 (+) + protamine condition (Figure 7), aggregation was promoted by the entanglement of macromolecules constituting the cell walls and membranes.

## 5. Conclusions

In this study, we proposed a new method of cotreatment with protamine and calcium hydroxide, an existing intracanal medication, and evaluated its efficacy against *E. faecalis* disinfection. Our results showed that complete sterilization is now possible, even in an alkaline environment below pH 11.5, which is considered necessary for *E. faecalis* sterilization. Although more detailed studies are needed for clinical applications, such as the evaluation of biofilm formation and verification of deep reachability in the dentinal tubules, we believe that this approach has the potential to provide a novel control method against *E. faecalis* for root canal treatment.

## Figures and Tables

**Table 1:** MIC and MBC of protamine against *E. faecalis*.

MIC [μg/mL]	MBC [μg/mL]
250	>1000

## Data Availability

The data supporting this study’s findings are available from the corresponding authors upon reasonable request.
